# Clinical Validation of Dental Implant Stability by Newly Designed Damping Capacity Assessment Device during the Healing Period

**DOI:** 10.3390/medicina58111570

**Published:** 2022-10-31

**Authors:** Ho-Kyung Lim, Sung-Jae Lee, Yujin Jeong, Jong-Seok Lee, Jae-Jun Ryu, Ji-Suk Shim, In-Seok Song

**Affiliations:** 1 Department of Oral & Maxillofacial Surgery, Korea University Guro Hospital, Seoul 08308, Korea; 2 Department of Oral & Maxillofacial Surgery, Korea University Anam Hospital, Seoul 02841, Korea; 3 Department of Biostatistics, Korea University College of Medicine, Seoul 02841, Korea; 4 Department of Prosthodontics, Korea University Anam Hospital, Seoul 02841, Korea; 5 Department of Prosthodontics, Korea University Guro Hospital, Seoul 08308, Korea

**Keywords:** dental implants, stability, dentistry, analytic device

## Abstract

*Background and Objectives:* To evaluate the stability of a dental implant and the effectiveness of a newly designed damping capacity assessment device by improving the number of blows and strength evaluated by a prospective clinical study. *Materials and Method:* The stability of dental implants was measured in 50 implants in a total of 38 patients. Measurements were performed using Anycheck and Periotest M devices, twice in total, divided into buccal and lingual directions. In addition, measurements were performed on the day of surgery, two weeks, one month, two months, and three months after surgery for a total of five times. After the standardization of the measured values, the differences and changes over time for each device were observed. *Result:* No difference in standardized values between the two devices was observed at any time point. In both devices, stability decreased at two weeks postoperatively but gradually increased thereafter. No differences were observed in the values according to the measurement direction. *Conclusions:* The damping capacity of Anycheck was similar to that of Periotest M. After a slight decrease in stability two weeks after implant placement, implant stability increased over time.

## 1. Introduction

Osseointegration of dental implants is affected by various factors such as the type of implant surface, density of the alveolar bone, age of the patient, whether or not a bone grafting is performed, and the volume of the alveolar bone [1]. Various methods of measuring the stability of dental implants have been used in clinical practice. The insertion torque value measurement method, such as the Osstell method using resonance frequency analysis (Osstell device, Integration Diagnostics AB, Sävedalen, Sweden), and Periotest M method using damping capacity assessment (Periotest M device, Gulden Messtechnik, Bensheim, Germany) have been widely used [2,3,4]. Each measurement method has its characteristics. Osstell is a non-contact measurement method, and its measurement values are internationally standardized. However, a separate measuring device (Smartpeg) is required, and there is a risk and inconvenience in releasing the healing abutment for the measurement. Periotest M is convenient and safe for measuring the stability of a healing abutment. However, the measured value is affected by the angle of impact and the high strength of the blow, and the number of blows is rather high (16 times) causing a feeling of rejection in the patient.

The recently developed modified damping capacity measuring instrument (Anycheck, Neobiotech Co., Ltd., Seoul, Korea) has high reproducibility, and it is possible to directly contact the measurement target by improving the hitting method [5]. The number of measurements was also reduced to six, and when the stability measurement was less than 70, the function of hitting the implant was decreased to two times to reduce the impact on the implant. There are several in vitro and animal tests, but there are still few studies on their effectiveness in clinical practice [5,6].

In this clinical study, the stability of implants during the healing period was verified using this new damping capacity assessment device. In addition, the similarity of the measured values was evaluated and compared with that of the existing Periotest M equipment.

## 2. Materials and Methods

Patients who visited the Korea University Anam Hospital from January 2020 to December 2021 and who had healing abutments placed after implant placement under local anesthesia were included in the study. The following patients were included in the study: those who planned to have a dental implant and healing abutment placed on the day of surgery and those who were older than 19 years who had a firm willingness to participate in this study and eventually agreed to participate in the study. Patients were excluded if the implant was replaced due to previous failure, placed immediately on the same day after tooth extraction, or if the procedure included a large amount of vertical augmentation of the alveolar bone or sinus grafting due to severe bone loss. A total of 38 patients with 50 implants were included in this study. This prospective clinical study was conducted with the approval of the Institutional Review Board of Korea University Anam Hospital (No. 2020AN0105).

Implant-first surgery was performed under block or infiltration anesthesia using 2% lidocaine epinephrine (1:100,000 epinephrine containment) in the outpatient clinic. If bone defects, such as dehiscence, existed, bone grafting using xenografts (BioOss, Geistlich Pharma AG, Zürich, Switzerland) was performed simultaneously with implant placement. Only bone level and internal hex connection fixtures (LUNA, Shinhung Co., Ltd., Seoul, Korea; ISⅡ or ISⅢ, Neobiotech Co., Ltd., Seoul, Korea) were used for implant placement. The healing abutment was placed after implant placement, and if the incision was previously made, sutures were made using nylon without tension. Implant stability was measured as previously described. After pressure dressing with a sterile gauze bite was performed, postoperative caution was explained to the subjects. An antibiotic (cephalexin 1000 mg, t.i.d.) and a non-steroidal anti-inflammatory agent (zaltoprofen 80 mg, t.i.d.) were prescribed for 5 days, and 0.12% chlorhexidine solution mouth rinse was administered daily.

**Figure 1:**
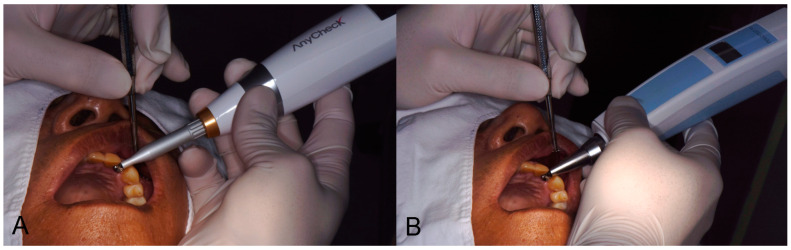
Clinical application of Anycheck and Periotest M equipment. (**A**) Anycheck, (**B**) Periotest M).

Implant stability was measured twice each on the buccal (labial) and lingual (palatal) sides using two different damping capacity analysis devices (Periotest M, Anycheck). The stability value measured by Periotest M is referred to as the Periotest value (PTV), ranging from −8.0 to +50.0, which is closer to −8.0% when the material has more rigidity. It was measured through 16 tapping motions. The value of the implant stability test (IST) measured by Anycheck was designed to be similar to the implant stability quotient value (ISQ scale), ranging from 0 to 100, and was measured through six rounds of slight tapping motion. The IST value was then calibrated according to the height of the healing abutment according to the manufacturer’s instructions: no calibration at 4 mm height, +2 per 1 mm shorter, and −2 per 1 mm longer than the height of the healing abutment. When both devices are driven at a point 2–3 mm away from the healing abutment, the stability value is derived through effective hitting (Figure 1).

**Figure 2:**
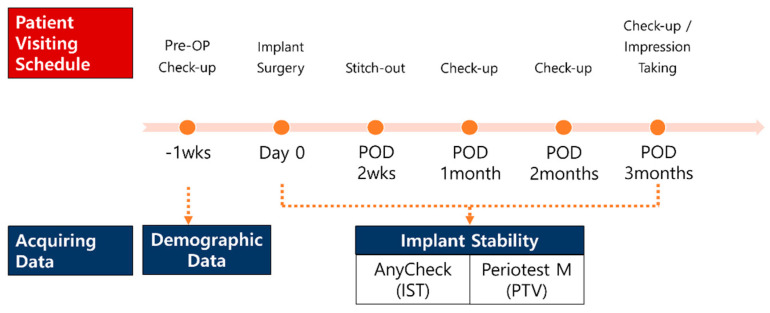
Schematic diagram of the clinical trials.

The participants were instructed to visit the clinics at 2 weeks, 1 month, 2 months, and 3 months after the first implant surgery. At each follow-up, stability measurements were performed in the same manner as on the operative day. After 3 months, the patients were referred to the prosthodontic department for implant prosthesis restoration, if no major complications occurred, or additional follow-ups were arranged if the stability value was considered insufficient to be loaded (Figure 2). In addition to implant stability, implant sites, type of fixtures, diameter and length of the fixtures, the gingival height of the healing abutments, and bone grafting were recorded at all follow-up periods.

For statistical analysis, the implant stability measured by damping capacity analysis devices after implant placement was evaluated using covariance analysis of repeated measurements implemented using SAS Proc Mixed (SAS Institute, Inc., Cary, NC, USA). The device (Periotest M or Anycheck) was the between-subject factor and time (on the day of surgery, 2 weeks, 1 month, 2 months, 3 months) was the within-subject factor. MEAN/SD is the observed mean and standard deviation and LSMEAN/SE is the predicted mean and standard error of the statistical model. The scales of the two measurement devices were standardized using the Z-score standardization method. Statistical significance was set at *p* < 0.05. Analyses were performed for the buccal (labial) side, lingual (palatal) side, and total values. Statistical analysis was performed using the Statistical Analysis System version 9.4 (SAS Institute, Inc., Cary, NC, USA).

## 3. Results

The characteristics of the participants and the implants are presented in Table 1. The mean age was 66 years, and 20 men and 18 women were included in the study. Twenty-three implants were placed in the maxilla, 27 in the mandible, 4 in the anterior region, and 46 in the posterior region. Implant fixtures from the following three manufacturers were used: LUNA (Shinhung, Seoul, Korea), 22; ISII (Neobiotech, Seoul, Korea), 20; and ISIII (Neobiotech, Seoul, Korea), 8. For the fixture size, six short implants and 49 regular implants were used. For the height of the healing abutment, 4 mm was used the most (26 pieces). Bone grafting was performed on 21 patients.

**Figure 3:**
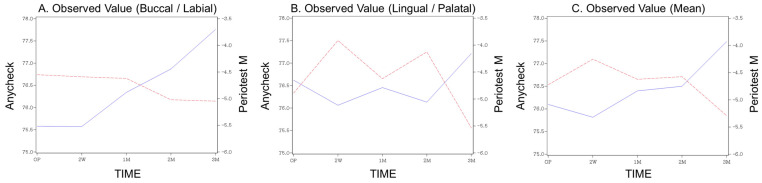
The observed values over time. (**A**) Buccal, (**B**) lingual, (**C**) mean.

**Figure 4:**
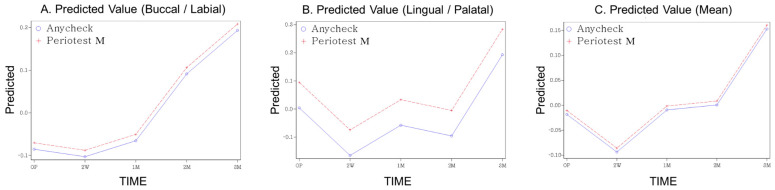
The predicted value over time (Z-score standardized, (**A**) buccal, (**B**) lingual, (**C**) mean).

The mean values and standard deviations of measured stability at each follow-up period are presented in Table 2. When stability was measured using Periotest M, the average stability immediately after surgery decreased at two weeks but gradually increased thereafter, showing overall higher stability at the end of three months than immediately after surgery. In the case of Anycheck, similar to Periotest M, the average of the measured values decreased in the second week after the operation, but gradually increased thereafter and showed higher stability than immediately after the operation from one month. (Table 2, Figure 3). This trend was similar for the buccal, lingual, and average scores. Contrary to the pattern of the measured values, both the standardized Z-scores of Periotest M and Anycheck showed a significant increase with time after a decrease at two weeks postoperatively (Table 3, Figure 4) (*p* < 0.0001). This is illustrated in Figure 4. This trend was significantly observed in the buccal, lingual, and average areas. It is observed that the “stability dip” is formed between two weeks and one month after implant placement, and the stability increases rapidly as it reaches the third month. At all time points, no difference in the standardized values was observed between the two instruments, Periotest M and Anycheck (*p* > 0.01).

## 4. Discussion

The results of this study showed that the damping capacity of Anycheck at all time points and in all hitting directions showed a tendency similar to that of Periotest M. Although the measured values were different, in the corrected values, the results of the two instruments were almost identical. In addition, after a slight decrease in stability two weeks after implant placement, implant stability increased over time, and both devices showed a significant difference with time.

Currently, the most widely used devices for measuring dental implant stability are Osstell, which can measure ISQ values based on resonance frequency analysis, and Periotest M, which is based on damping capacity assessment, as mentioned in the introduction [7]. The advantage of Osstell is that there is no tapping of the implant during measurement; therefore, there is less discomfort for the patient. However, for each implant product, a smart peg with a matching inner surface must be provided, and the smart peg fastening process may affect the fixation of implants with weak initial stability [8,9]. In the case of Periotest M, there is no such connection process, but the blow is strong and the number of blows is relatively large (16), which can cause patient discomfort, and the measured value can be affected by the blow angle [10,11].

The Anycheck device is an improved version of these two devices. It does not require a superstructure connection process such as Osstell for measurement, and the strength and frequency of blows have been dramatically improved compared with Periotest M [5]. In addition, to increase the user’s intuition, it is displayed differently in red, orange, and green depending on the range of the measured value, in order that stability can be recognized without reading the number [5]. Therefore, the Anycheck device makes it easier to measure implant stability than existing devices. However, despite having a wider effective striking angle than Periotest M, it can only be measured when the striking angle is in the range of 0° to 30° from the ground, and the final result value must be corrected because the resulting value may vary depending on the length of the healing abutment [6].

Implant stability is divided into two types: primary and secondary. Primary stability refers to the initial mechanical stability, which occurs because of friction through contact between the bone and implant surface [12]. If the initial fixation is insufficient and the micro-movement reaches a level exceeding 50–100 μm, osseointegration may be damaged, and as a result, tissues other than bone, such as fibrous tissue, may be formed around the implant [13]. Secondary stability refers to the stability of the biological form through bone regeneration and remodeling at the implant-tissue interface [14]. Differentiating osteogenic cells migrate to the implant surface to form a mineralized interfacial matrix around the implant and then undergo remodeling to complete osseointegration [15]. Total implant stability is composed of synthesizing this primary and secondary stability, and most of the studies on total implant stability report that the value decreased slightly immediately after implant placement and then gradually increased thereafter [16,17,18]. This pattern has been described as a drop or dip [19]. In one study, it was mentioned that this dip exists between two and four weeks using a mathematical model through curve-fitting [20], and a similar pattern of stability change was also observed in this study. At the second week after the operation, the measured values of both devices showed a decreasing pattern and then gradually increased thereafter. This means that the theoretical stability dip is also observed in actual clinical practice. Furthermore, this suggests the need to easily and conveniently measure implant stability in order that implants can be loaded at the right timing.

There are many studies on the comparison of Osstell, and Periotest M, which are existing devices for measuring implant stability, and the clinical similarity between the two devices has been verified to some extent [3,6,21,22,23,24,25,26]. The Anycheck device was developed relatively recently; therefore, there are not many studies using the Anycheck device. In particular, no clinical studies have yet been conducted. However, a high similarity between Anycheck and other devices can be observed consistently in existing studies and in this clinical study. In a validity analysis of an ex vivo study using porcine bones, a very high correlation was observed between the measured values of Anycheck, Osstell, and Periotest M, and a linear relationship between the insertion torque and the measured values was observed [6]. Similarly, in one in vitro study, a high correlation between the three devices was observed, and it was observed that the diameter of the healing abutment did not affect the measured value, unlike the healing abutment length, which affected the Anycheck measured value [5]. A previous study observed a linear correlation between the vibration frequency and the Anycheck value measured while controlling the peri-implant artificial bone level [27].

This clinical study has some limitations. Although Osstell and Periotest M showed almost equal reliability in numerous studies, Osstell was not applied in this study. However, if comparison with Osstell were performed, abundant results would have been derived. In addition, the fact that the effects of the jaw arch, implant specifications, and bone graft could not be controlled is another limitation of this study. Nevertheless, in the results, a similar and uniform tendency of the Anycheck device could be observed when compared with Periotest M, and the similarity along the timeline could also be observed; therefore, it is considered that the clinical significance of this study is sufficient. Through this prospective clinical study, the new damping assessment device with reduced patient discomfort and high clinical versatility suggested the possibility of clinical replacement by showing implant stability measurements similar to those of existing equipment.

## 5. Conclusions

During the observation period of three months, the damping capacity of Anycheck showed a similar tendency to that of Periotest M. After a slight decrease in stability two weeks after implant placement, implant stability increased over time. Through this study, the clinical substitution potential of the Anycheck device, which has a simpler measurement method and equivalent implant stability measurement power, was observed.

## Figures and Tables

**Table 1:** Demographic data of the patients and characteristics of the dental implants.

Investigated Item	Number
Patients	38
Age, mean (range)	66 (36–89)
Sex	
Male	20 (53%)
Female	18 (47%)
Total implants	50
Jaw	
Maxilla	23 (46%)
Mandible	27 (54%)
Location	
Anterior	4 (8%)
Posterior	46 (92%)
Fixture (manufacturer)	
LUNA (Shinhung)	22 (44%)
ISⅡ (Neobiotech)	20 (40%)
ISⅢ (Neobiotech)	8 (16%)
Fixture (size)	
Length	
Short (<8.0 mm)	6 (12%)
Regular (8.0–11.5 mm)	43 (86%)
Long (>11.5 mm)	1 (2%)
Diameter	
Narrow (≤3.5 mm)	1 (2%)
Regular (4.0–5.0 mm)	49 (98%)
Wide (>5.0 mm)	0
Healing abutment (GH)	
3 mm	4 (8%)
4 mm	26 (52%)
5 mm	12 (24%)
6 mm	7 (14%)
7 mm	1 (2%)
Bone grafting	21 (42%)

**Table 2:** The mean value and standard deviation of measured stability at each follow-up period.

Device	Post-op Period	Mean	SD
Periotest M	Op	−4.72	2.92
	2 W	−4.25	4.37
	1 M	−4.62	3.50
	2 M	−4.57	3.34
	3 M	−5.29	2.84
Anycheck	Op	76.10	6.89
	2 W	75.82	9.87
	1 M	76.40	8.42
	2 M	76.50	7.85
	3 M	77.48	6.92

Abbreviation: Op, operation day; 2 W, post-operative 2 weeks; 1 M, post-operative 1 month; 2 M, post-operative 2 months; 3 M, post-operative 3 months.

**Table 3:** The implant stability measured by damping capacity analysis devices after implant placement.

	Tapping Location	Mean	SD	LSMEAN	SE	*p*-Value
Periotest M	both	0.000	0.999	0.04322	0.07911	0.6626
Anycheck		0.000	0.999	−0.0030	0.07911	
OP		−0.014	0.861	−0.014	0.06071	<0.0001
2 W		−0.090	1.219	−0.107	0.08685	
1 M		−0.005	1.025	−0.037	0.07259	
2 M		0.005	0.942	0.028	0.07063	
3 M		0.157	0.837	0.231	0.06275	
Periotest M	Buccal	0.000	1.000	0.021	0.1192	0.9275
Anycheck		0.000	1.000	0.006	0.1192	
OP		−0.078	0.933	−0.078	0.09283	0.0325
2 W		−0.072	1.172	−0.096	0.1178	
1 M		−0.016	1.061	−0.058	0.1053	
2 M		0.086	0.875	0.099	0.08901	
3 M		0.148	0.861	0.201	0.09207	
Periotest M	Lingual	0.000	1.000	0.066	0.1058	0.5173
Anycheck		0.000	1.000	−0.024	0.1058	
OP		0.049	0.782	0.049	0.07777	0.0031
2 W		−0.108	1.270	−0.120	0.1278	
1 M		0.006	0.995	−0.012	0.0998	
2 M		−0.076	1.003	−0.050	0.1093	
3 M		0.165	0.819	0.238	0.08636	

Abbreviation: Op, operation day; 2 W, post-operative 2 weeks; 1 M, post-operative 1 month; 2 M, post-operative 2 months; 3 M, post-operative 3 months. Device (Periotest M or Anycheck) was the between-subject factor and time (OP, 2 W, 1 M, 2 M, 3 M) was the within-subject factor. MEAN/SD is the observed mean and standard deviation and LSMEAN/SE is the predicted mean and standard error of the statistical model. The scales of the two measurement devices were standardized using the Z-score standardization method. Statistical significance was set at *p* < 0.05.

## Data Availability

The datasets are available from the corresponding author on reasonable request.
